# Changes in Trait Mindfulness after a Brief Mindfulness Training Program of Self-Breathing

**DOI:** 10.3390/healthcare12202019

**Published:** 2024-10-11

**Authors:** Momoe Sakagami, Tomoe Yokono, Hansani Madushika Abeywickrama, Nao Seki, Michio Miyasaka, Mieko Uchiyama

**Affiliations:** 1 Department of Nursing, Graduate School of Health Sciences, Niigata University, 2-746 Asahimachi, Niigata 951-8518, Japan; tyokono@clg.niigata-u.ac.jp (T.Y.); hansani@clg.niigata-u.ac.jp (H.M.A.); nao@clg.niigata-u.ac.jp (N.S.); miyasaka@clg.niigata-u.ac.jp (M.M.); uchiyama@clg.niigata-u.ac.jp (M.U.); 2 Department of Nursing, School of Health Sciences, Faculty of Medicine, Niigata University, 2-746 Asahimachi, Niigata 951-8518, Japan

**Keywords:** brief-mindfulness training, trait mindfulness, Five-Facet Mindfulness Questionnaire, self-breathing practice

## Abstract

Background: Developing and cultivating mindfulness exerts a positive effect on psychological and cognitive performance. Sharpening the skill requires continuous mindfulness-based training (MT), which can be challenging for people leading busy lives. Therefore, the current study examined whether trait mindfulness can be improved by a flexible and brief MT program of self-breathing using a pre–post intervention design. Methods: Trait mindfulness was assessed using the Japanese version of the Five Facet Mindfulness Questionnaire (FFMQ) before the intervention (pre), after 2 weeks (during), and 4 weeks after the intervention started (post). Data were analyzed using the Friedman test followed by the Dunn–Bonferroni correction. Results: The study sample consisted of 22 healthy participants aged from 20 to 60 years with no previous experience with yoga or meditation equivalent to MT. The mean number of days of MT practice was 26.4, and 11 participants had interruptions. The median values of pre-, during-, and post-total FFMQ scores were 115.5, 123, and 129, respectively. Significant differences were observed in the total pre and post (*p* < 0.001) and during and post (*p* = 0.002) FFMQ scores, though a medium effect was found (r = 0.30) only between the pre and post scores. Of the five sub-scales of FFMQ, significant differences were observed only between pre and post Observing (*p* = 0.01), Nonreactivity (*p* < 0.001), and Describing (*p* = 0.01), and during and post Nonjudging (*p* = 0.016), and Nonreactivity (*p* = 0.025). Conclusions: Our findings suggest that the simple, brief, and flexible self-breathing method employed in this study has a substantial effect on fostering trait mindfulness and, therefore, can be adopted by people with hectic daily schedules.

## 1. Introduction

Mindfulness is defined as “the awareness that emerges through paying attention on purpose, in the present moment, and non-judgmentally to things as they are [1] (p. 47)”. It is “a skill of particularized attention” to avoid making judgments about one’s experience in the present moment [2] (p. 68). This attention skill leads to the promotion of self-perception at the cognitive level and the improvement of insight, encouraging one to see his/her own thoughts as temporary mental events [3] (pp. 129–142). Further, it promotes a healthier way of engaging with internal experiences without changing or escaping emotional acceptance [4]. It has been found to be a useful skill for treatment in medical settings and for people navigating a stressful society [5]. Cultivating mindfulness has been scientifically shown to bring various positive effects, such as pain management, recovery from addictions, stress reduction, and enhancement of the effectiveness of psychotherapy [6,7,8,9]. Further, several meta-analyses provide evidence for the association between mindfulness and improvements across a wide range of well-being measures, including cases of depression, anxiety, distress, and coping [10,11,12].

In order to cultivate mindfulness, it is essential to engage in continuous training, as mindfulness is often compared in mindfulness teachings to a muscle that can be strengthened through practice [13]. The training involves consciously observing the mind and body and repeatedly practicing the process of accepting experiences as they are from one moment to the next [14] as an accumulation of mindfulness-based training (MT). This is exemplified by the scientifically validated Mindfulness-Based Stress Reduction (MBSR) program, a systematic eight-week program consisting of regular weekly guiding sessions of 2.5 h and continuous daily self-practice of 45 to 60 min. Further, in the first to second week, the program includes body scans where participants pay attention to and sense their own bodily sensations, and in the third to fourth week, they practice yoga, focusing on the sensations that arise while assuming postures. However, despite its proven effectiveness, it is difficult to incorporate a systematic program over a long period into daily life. In particular, for people who do not require clinical intervention, who work irregular hours, or whose social roles make having time to spare difficult, integrating and continuing such a time-consuming training may be too challenging [15].

Recent studies have shown that brief MT programs improve mental health, reduce depressive tendencies [16,17], enhance mood [16,17,18], and improve the ability to remain present without avoiding unpleasant feelings and bodily sensations [19]. Additionally, practices lasting 30 min or less per session have been shown to reduce anxiety and perceived stress, improve mindfulness, lower blood cortisol levels, and alter the gray matter of the brain to protect against mood-related disorders and age-related cognitive decline [20,21,22]. Brief MT sessions of 4 h or less for healthcare professionals have also shown potential for improving their well-being. However, the effects of such practice on mindfulness have not been consistent. In addition, a decrease in participant numbers has been observed even in brief MT programs (5–20 min) with multiple sessions [15]. Although there is evidence that higher levels of trait mindfulness, which reflects an individual’s general mindfulness level, may be linked to health benefits [6], it remains unclear whether brief or short-term programs can effectively alter an individual’s trait mindfulness.

We, therefore, propose a brief mindfulness-based self-training program called the self-breathing method, considering the continuity of practice for people who find it challenging to maintain a continuous practice due to their occupations or social roles. The self-breathing method is a new MT program (Table 1), and its features include focusing on a single type of MT practice, brief sessions of 10 min, designating the entire practice period as self-care, providing explanations only before the start of the intervention, allowing practitioners to choose the location and timing of their convenience to practice, and accepting intermittent practice from the beginning. Through these measures, our goal was to design a simple, brief, and flexible program that takes into account the physical and psychological burdens of the practice on practitioners. The purpose of this study was to examine changes in trait mindfulness after a short period of continuous practice of the proposed self-breathing method.

## 2. Materials and Methods

### 2.1. Study Design

We applied a pre–post comparison study design. The study period was from October 2021 to March 2023.

### 2.2. Participants

The inclusion criteria for participants were as follows: adult males and females aged 20 years or older and younger than 60; people who had no prior continuous practice experience of yoga or meditation; people who had no cognitive, perceptual, or motor impairment at the time of recruitment; people who did not have any physical or mental health problems that had been diagnosed or treated; and people engaged in academic or occupational activities on weekdays. The exclusion criteria were continuous practitioners of yoga and/or meditation to minimize selection bias. Participants recruited voluntarily posters and snowball sampling [23] (p. 292). We utilized snowball sampling, also referred to as network sampling, to achieve the desired sample size due to difficulties faced in recruiting study participants due to the COVID-19 pandemic. In this sampling method, early participants were asked to share the posters and introduce the study to their acquaintances.

### 2.3. Sample Size

The required sample size was calculated using the free software G*Power 3.1.9.6 (Institute of Experimental Psychology, Heinrich-Heine-University: HHU) for power analysis and the F-test (repeated measures ANOVA). The significance level was set at 0.05, the power was 80%, and the effect size was 0.25, which is the moderate effect size typically observed in mindfulness-based interventions through self-care [24]. The calculation yielded a sample size of 28 participants.

### 2.4. Data Collection and Tools

#### 2.4.1. Basic Attributes

The basic characteristics of the participants collected were age, sex, and occupational status. In addition, subjective stress at the baseline was assessed by asking the participants to rate their stress level during the 7 days before the inquiry using a five-point Likert scale.

#### 2.4.2. Trait Mindfulness

To measure trait mindfulness, we used the Japanese version of the Five Facet Mindfulness Questionnaire (FFMQ). The FFMQ is a scale developed by Bear et al. (2006) [25]. It is a 39-item scale with a 5-factor structure that measures the complex ability of mindfulness in the following aspects: Observing, Nonreactivity, Nonjudging, Acting with awareness, and Describing. Responses are rated on a five-point Likert scale from “not at all true” to “always true”, with total scores ranging from 39 to 195. Higher scores indicate higher mindfulness, except for reverse-scored items. The Japanese version of the FFMQ was developed by Sugiura et al. and has been shown to have psychometric properties comparable to those of the original FFMQ. It had a good internal consistency for the total score (α = 0.80) and the five subscales (α = 0.67–0.85). Construct validity has been confirmed through predictive correlations with related scales, establishing it as a reliable and valid tool for use in Japan. [26]. The meanings of the five factors of FFMQ are as follows: Observing, being aware of and paying attention to internal and external experiences, such as sensations, cognitions, emotions, visual stimuli, sounds, and smells; Nonjudging, a non-judgmental attitude toward sensations, cognitions, and emotions; Nonreactivity, an attitude of allowing thoughts and emotions to be as they are without trying to react to or push them away; Acting with awareness, being conscious of the action being taken in the present moment; and Describing, verbalization of internal experiences [25,26].

### 2.5. Study Protocol

**Figure 1:**
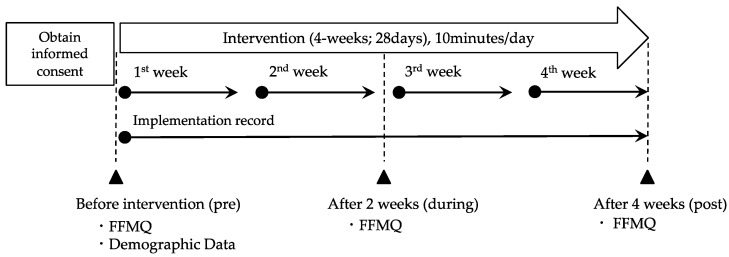
Study protocol. The intervention consisted of a 10-minute daily self-breathing practice over four weeks. Basic attributes (age, sex, occupation, and subjective stress level) data were collected at the baseline, while FFMQ scores were measured before (pre) the intervention, after 2 weeks (during), and 4 weeks (post) after the intervention started. Participants recorded the date and time of their training throughout the intervention period, and implementation records were collected at the end of the intervention. FFMQ, the Japanese version of the Five Facet Mindfulness Questionnaire. MT, mindfulness-based training.

The study protocol is illustrated in Figure 1.

Basic attributes were measured at the baseline, while trait mindfulness was measured at three points: before the intervention (pre), after two weeks (during), and after four weeks, at the end of the intervention (post).

Participants were asked to record the date and timing of their training sessions over the 4-week period on a log sheet to monitor the intervention’s implementation status.

### 2.6. Intervention: Self-Breathing Method (Table 1)

The intervention is called the ‘self-breathing method’, and involves a short self-mindful breathing practice (Table 1).

In this practice, an individual focuses attention on his/her own breathing while concentrating on what happens during the process. This has been rated the simplest and most effective method of mindfulness practice [1,27,28]. Following the typical mindfulness instructions provided in MBSR, we explained the practice method to participants as follows:

(1) Pay attention to the natural sensations of breath in the body.
(2) Follow the breath through full cycles, from the beginning of an inhalation to the end of an exhalation and continuing to the next cycle.
(3) If your mind wanders to internal and external stimuli and you lose awareness of the breath, gently return your attention to breathing.

We created pamphlets and an explanatory video to ensure uniformity in the description of the practice method and to try to avoid misinterpretation of the instructions.

Additionally, we made the explanatory video available on the web and provided participants with access to it so that they could freely review the practice method during the intervention period. The practice time per session was set to be equivalent to one-fifth of the daily MT self-practice time recommended in MBSR. Although the exact dose–response relationship between MT practice time per session and mindfulness is presently unclear, it has been suggested that the state of mindfulness improves equally with 20-minute and 10-minute mindfulness meditation, and it is independent of session duration [29].

### 2.7. Analysis

Basic attributes were reported as frequencies and percentages, while total and sub-factor FFMQ scores for the three time points were presented as median and interquartile range (IQR) values. Data were analyzed using non-parametric tests as the sample size was small and skewed distributions of data were observed.

Participants completed the four-week intervention either consecutively or intermittently (with interruptions). For analysis, participants were categorized into two groups: those who completed the four-week intervention period or interrupted for less than three days (continuous group) and those with interruptions of three days or more (intermittent group). The primary outcome was the overall change in trait mindfulness (total FFMQ score), and the secondary outcome was the change in each of the five mindfulness skills represented by the FFMQ sub-factors.

The Friedman test, followed by the Dunn–Bonferroni method, was used to examine differences in FFMQ scores between the three time points (pre–post, pre–during, and during–post). The Mann–Whitney U test was used to compare the post-intervention total FFMQ scores and the during–post changes in sub-factor scores between the continuous and intermittent groups. In addition, to measure the strength of the associations, the effect size was calculated using Pearson’s product–moment correlation coefficient (r). Effect size is a standardized index independent of sample size that ranges from 0 to 1. The greater the absolute value, the larger the effect size, with the following judgment criteria: 0.1 indicates a small effect, 0.3 a medium effect, and 0.5 a large effect. The formula used is r = Z/√n, where the test statistic is converted into Z [30,31].

Statistical analyses were performed using the IBM Statistical Package for Social Sciences (SPSS) (Ver. 28.0.1.0., IBM Corp., Armonk, NY, USA), with *p*-values of lower than 0.05 being considered statistically significant.

### 2.8. Ethical Considerations

The study was approved by the Ethics Committee of Niigata University (Ethical Approval number 2021-0120). All participants provided written informed consent.

## 3. Results

### 3.1. Recruitment and Selection of Participants (Figure 2)

The participant recruitment period was from October 2021 to December 2022. Twenty-five applicants met the inclusion criteria; however, one withdrew after receiving the explanation, and two did not complete the study because they did not respond to the FFMQ in week 2. Thus, the final analysis included twenty-two participants. Of these, eleven participants completed the intervention with interruptions, with six interrupting it for less than three days and five for more than three days.

**Figure 2:**
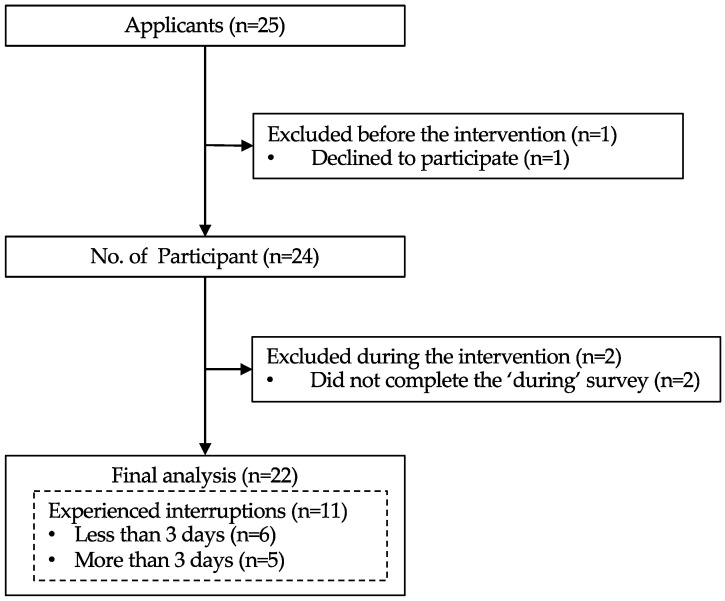
Diagram depicting participant recruitment, exclusion, and analysis.

### 3.2. Basic Attributes of Participants

The majority of the participants were female (n = 20) and were aged between 20 and 29 years (n = 16), while thirteen of them were university students. Eight participants rated the subjective stress level as ‘high or somewhat high’ (Table 2).

The mean number of days of MT practice was 26.4. Six and five participants interrupted the intervention for 1–2 days and 4–7 days, respectively (Table 3). Among those with interruptions of 4 to 6 days, four participants cited lack of time as the reason for the intermittent practice, while one participant with a 7-day interruption cited fatigue.

### 3.3. Changes in Trait Mindfulness

**Figure 3:**
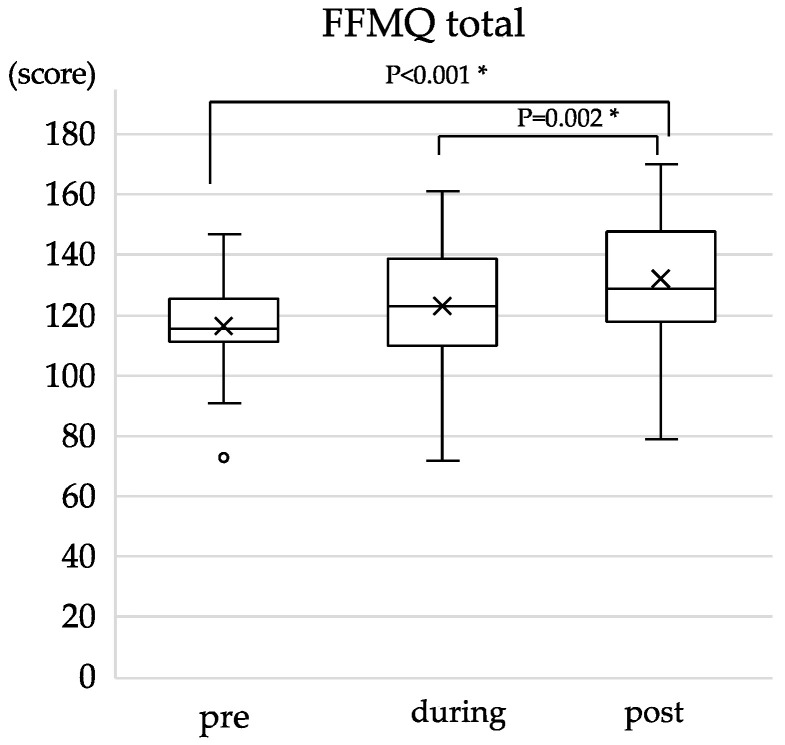
Changes in total FFMQ scores before (pre), during, and after (post) the intervention (n = 22). FFMQ, the Japanese version of the Five Facet Mindfulness Questionnaire. The cross marks and the horizontal line indicate the mean and the median, respectively. The range of the total score is 39–195. The asterisk indicates a *p*-value lower than 0.01 (*p* < 0.01).

**Figure 4:**
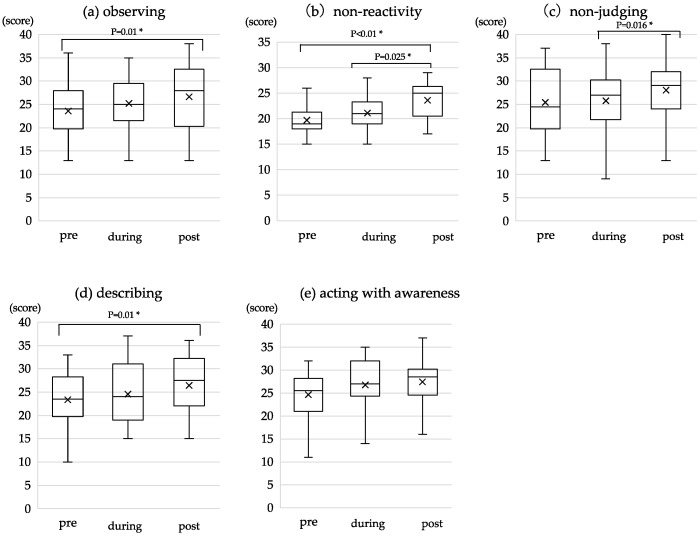
Change in FFMQ sub-scores during the intervention period (n = 22). Changes in scores for (**a**) Observing, (**b**) Nonreactivity, (**c**) Nonjudging, (**d**) Describing, and (**e**) Acting with awareness. Pre, before the intervention; during, two weeks after the start of the intervention; post, four weeks after the start of the intervention. The cross marks and the horizontal line indicate the mean and the median, respectively. The asterisk indicates a *p*-value lower than 0.05 (*p* < 0.05). The range of scores is as follows: (**a**) Observing (8–40), (**b**) Nonreactivity (7–35), (**c**) Nonjudging (8–40), (**d**) Describing (8–40), and (**e**) Acting with awareness (8–40).

The median and IQR of the total FFMQ and sub-factor scores at the three time points are presented in Table 4. There were significant differences between pre and post (*p* < 0.001, r = 0.30) and between during and post (*p* = 0.002, r = 0.22) total FFMQ scores, indicating a significant change in trait mindfulness over time (Figure 3), with a medium effect observed only for the pre and post difference. Among the sub-factors of FFMQ, significant differences were observed between pre- and post-scores for Observing (*p* = 0.010, r = 0.19), Nonreactivity (*p* < 0.001, r = 0.27), and Describing (*p* < 0.010, r = 0.19). In addition, there was a significant difference between the during and post scores for Nonjudging (*p* = 0.016, r = 0.18) and Nonreactivity (*p* = 0.025, r = 0.17). Despite reaching statistical significance, the associated effect sizes for these differences were small. As for Acting with awareness, no significant differences were observed between the scores at any of the three points, indicating no detectable change in trait mindfulness in the study sample (Figure 4).

Furthermore, between the continuous (n = 17) and intermittent (n = 5) groups, there were no significant differences in either the post-intervention total FFMQ score or the during–post change of scores for each sub-factor (Table 5). However, small to medium effect sizes were observed at each measurement time point.

## 4. Discussion

### 4.1. Changes in Trait Mindfulness through the Self-Breathing Intervention

This study examined changes in the trait mindfulness of participants using the self-breathing method, a new MT program. The results indicated that the total FFMQ score, which represents trait mindfulness, showed significant changes from baseline to post-intervention and from during- to post-intervention.

Mindfulness encompasses two aspects: state mindfulness and trait mindfulness [32]. State mindfulness refers to a temporary state of an individual, such as “being attentive at a specific moment”, whereas trait mindfulness describes the daily characteristics of a stable individual, such as “being consistently attentive”. Although closely related, state mindfulness, when practiced repeatedly, can develop into trait mindfulness, a lasting mental characteristic [33,34]. As state mindfulness becomes more stable and idiosyncratic, it reflects a personality trait that emphasizes an individual’s ability to remain aware of and attentive to their experience in the present moment [32].

To date, no dose–response relationship between mindfulness and MT has been established, either concerning the duration of a single practice session or the cumulative time of practice. However, it has been shown that the state of mindfulness can be improved by a brief single MT session [29] and that the development of trait mindfulness is related to the amount of MT practice using meditation [35,36]. In the present study, changes in trait mindfulness between the start of the intervention and two weeks later (during the intervention) did not attain statistical significance (*p* = 0.683, r = 0.08). However, significant differences were found during the periods from baseline to post (*p* < 0.001, r = 0.30) and from during to post (*p* = 0.002, r = 0.22), with a medium effect size observed for the pre- and post-intervention difference. This suggests that each MT using the self-breathing method repeatedly evoked the preliminary mindful state necessary for developing a lasting mental characteristic and that continued practice beyond two weeks likely contributed to accumulating sufficient MT practice to develop trait mindfulness. The differences between the subgroups of continuous and intermittent participants from during- to post-intervention were not significant, indicating that continuing practice with the self-breathing method for more than two weeks, even if intermittent to some extent, may satisfy the amount of MT practice that leads to the development of trait mindfulness. Furthermore, no statistically significant difference was found in the overall score between the intermittent and continuous groups, and the effect size was small, indicating that increasing the number of subjects is unlikely to result in a significant difference.

Cebolla et al. (2017) showed that the overall development of mindfulness is more related to the frequency and cumulative time of practice rather than the length or type of a single MT session [36]. Therefore, a brief MT program with methodological flexibility, like the self-breathing method adopted in the current study, should contribute to cultivating mindfulness traits, even with some interruptions.

### 4.2. Changes in Facets of Mindfulness through the MT of Self-Breathing Method

Following the 4-week intervention based on the self-breathing method, significant differences were found between the pre- and post-total FFMQ scores and the scores for all FFMQ sub-factors except for Acting with awareness from baseline to post. Differences in the rate of trait improvement among the FFMQ sub-factors were reported in a previous study using MBSR [35]. We observed changes in four sub-factors at the end of the intervention: Observing, Nonreactivity, Nonjudging, and Describing. Of these, Observing, Nonreactivity, and Nonjudging have been shown to change relatively early, within the first three weeks of MBSR practice [35], which is consistent with the changes observed during the four weeks of practice with the self-breathing method in the present study. Moreover, these three facets are considered useful in reducing various unpleasant symptoms and improving psychological functioning in mindfulness practice [37,38] and essential and central skills for developing trait mindfulness [38]. These findings suggest that the self-breathing method is a practice that contributes to the development of these three important mindfulness sub-factors that form the basis of overall trait mindfulness.

On the other hand, no changes were observed in the score for Acting with awareness during the intervention period with the self-breathing method, whereas it shows changes at a relatively early stage in an 8-week course in MBSR [35]. This lack of change may be attributed to the simplified nature of the self-breathing method, which involved shorter MT sessions and a focus on a single practice type. In MBSR, changes in Acting with awareness tend to be observed through the combination of multiple MT practices accumulated over about three weeks. In contrast, the self-breathing method followed the method of focusing attention on the breath as an anchor and used only one type of MT practice overlapping with concentration meditation [39], where practitioners narrow the scope of attention, concentrate on a single object or event, such as the sensation of breathing, and return their attention to it when distracted by external stimuli or internal thoughts. A previous study reports that the duration of meditation practice and lifetime practice experience are related to the development of Acting with awareness [38]. Therefore, as the self-breathing method was an intervention program consisting of only brief and simple practices, the failure of the observed changes to attain statistical significance may have resulted from insufficient practice time and inadequate accumulation of experience to develop Acting with awareness in about four weeks of practice. Therefore, we believe that there is room for consideration of the current program as practical content or to set the length of practice for developing conscious behavior.

Lastly, the sub-factor Describing has been observed not to change significantly in the early stages of the practice of MBSR [35,40]. This is thought to be because MBSR is a practice that does not focus on verbally labeling or noting experiences at the moment [35]. In contrast, the self-breathing intervention of the current study, consisting of similar MT practices, led to changes in Describing during the period corresponding to the first half of the MBSR program. A previous study that compared several meditation practices reported that neither the duration, frequency, lifetime experience, nor type of meditation practice correlate with the development of Describing statistically significantly [38]. The observed changes in Describing suggest that either self-breathing may possess more powerful effects than previously suspected or that some statistical fluctuations were at work which favored a statistically significant result for our experiment.

In summary, while the self-breathing method may not uniformly improve the skills represented by the mindfulness sub-factors within a four-week intervention period, it was observed to be a practice that cultivates the three important mindfulness sub-factors that form the basis of overall trait mindfulness.

### 4.3. Applicability of the Self-Breathing Method for Individuals with Limited Time

In this study, we considered the self-breathing method (Table 1), examined its effectiveness based on FFMQ scores, and observed changes in overall trait mindfulness as well as in the sub-factors that contribute to its development. Furthermore, we found that the change in the total FFMQ score did not significantly vary between the continuous and intermittent groups, suggesting that the self-breathing method, although a brief MT program, is a practice that helps cultivate trait mindfulness when continued, even intermittently, over a certain period.

While the effectiveness of the systematic MBSR program is well-established, it traditionally consists of 26 h of sessions, including eight 2.5 h classes and one full-day class as a period deemed necessary to acquire the skills and autonomy in mindfulness practice [39]. Mindfulness practice is beneficial for the mental health of healthcare professionals or other professionals who work irregular hours, but the time required for training and practice poses a significant barrier [15]. It has also been suggested that adaptations with shorter practice times may be valuable for populations for whom longer time constraints could hinder their ability or willingness to participate [40]. Therefore, we believe that the self-breathing method, being a brief MT program, offers a viable alternative that facilitates the development of key mindfulness skills and improves mental health for individuals with limited time to spare. We also designed our protocol of self-breathing method with an emphasis on practitioners’ continuity, taking into account the physical and psychological burdens of practice.

Although the intervention period and methods vary, the median dropout rate in meta-analyses of mindfulness-based interventions (MBI) in clinical settings is 15.5% [41], and the mean rate of positive evaluation from the intervention group in studies using mindfulness as self-care is 63% [42]. In the present study, although two participants withdrew after the first half of the intervention period, more than 90% (91.6%) completed the MT practice to the end, even with intermittent interruptions, suggesting that the self-breathing method supports practitioners’ continuity.

Additionally, by limiting each practice session to 10 min, the self-breathing method reduced the time burden on participants. While no participants interrupted their practice for more than two consecutive days, 80% (n = 4) of those with three or more interruptions mentioned a lack of time as the reason. This indicates potential for further improvements to the program, such as shortening the practice time or modifying the practice content.

### 4.4. Strengths, Limitations, and Future Research

The strengths of this study lie in the confirmation of changes in participants’ trait mindfulness through an intervention based on the self-breathing method. It demonstrated that even a simple, flexible, and brief four-week MT program that takes into account the physical and psychological burdens on practitioners is effective in cultivating trait mindfulness to a certain extent. On the other hand, there are some limitations. First, the study design was a pre–post comparison without a control group. Although this approach allows for evaluating the intervention’s effects, the influence of psychological biases associated with the intervention cannot be denied. Since the intervention in this study was conducted during a period of significant social disruption due to the COVID-19 pandemic, even small changes in participants’ daily routines may have contributed to positive outcomes, potentially influencing the intervention’s effectiveness. The absence of a control group in the current study and the lack of evidence from a similar population outside the pandemic period make it difficult to assess the impact of these confounding factors. While the inherent subjectivity of pre–post comparison studies makes it difficult to eliminate observer subjectivity, individual differences in baseline trait mindfulness make intra-individual comparisons effective. Second, the study period coincided with the COVID-19 pandemic, when infection prevention measures, such as stay-at-home orders, were imposed in Japan. This resulted in the participation of fewer participants than expected. Further, the study sample predominantly consisted of females aged between 20 and 29 years pursuing higher education, which is not representative of the general population, thereby limiting the generalizability of the results. Third, the inclusion criteria for participants did not account for people who subjectively felt a lack of time, making it difficult to conclude whether the intervention would have the same effects on people who perceive themselves as having no time to spare. The absence of significant differences in the FFMQ total score or sub-factor scores between the intermittent and continuous groups at each measurement time, along with small effect sizes, indicates that the impact of the number of subjects on the results was small. However, the moderate effect sizes observed for the sub-factor Observing, despite the lack of statistically significant differences, suggest that significant differences could emerge by increasing the number of subjects. Nevertheless, the self-breathing method adopted in the present study shows potential as a program for cultivating trait mindfulness and contributes valuable insights to the limited literature on mindfulness. Future research could incorporate various occupation groups and consider other dependent variables (such as stress and anxiety) to further assess the effectiveness of the intervention studied here. In addition, we recommend future studies with larger sample sizes to clarify the intervention effects between the continuous and intermittent groups.

## 5. Conclusions

Changes in FFMQ scores over the intervention period indicated that the intervention based on the self-breathing method led to improvements in participants’ overall trait mindfulness and skills considered useful in mindfulness, represented by the sub-factors Observing, Nonreactivity, Nonjudging, and Describing. Small effect sizes were found before and after the intervention for all five sub-factors of mindfulness, and a medium effect was observed in the cultivation of trait mindfulness as a whole.

The results suggest that the self-breathing method with brief and flexible sessions can be useful in cultivating mindfulness, offering a feasible way to engage in continuous mindfulness practice for individuals with busy schedules or those facing time constraints.

## Figures and Tables

**Table 1:** Study intervention: self-breathing Method.

	Details
Pre-intervention instructions	Provide a detailed explanation before initiating the intervention (once before the first session).
Method of explanation	Provide explanations using the pamphlet and the video created by the research group.
Content of explanation	The pamphlet focused on the specifics of the practice, such as how to pay attention to the natural sensations of breathing and what to do if attention is diverted from the breath. It also emphasized the importance of continuing the practice, even intermittently, without self-evaluating its effectiveness. Additionally, the pamphlet included basic information about mindfulness: “Mindfulness is a state of mind in which attention is focused on the present moment deliberately. Recent studies have shown that continued mindfulness-based training has a positive impact on mental health”. The video served as a practical guide, visually demonstrating how to adopt the posture during practice, complementing the pamphlet explanation. It was available online for viewing at any time during the practice period, enhancing the learning experience.
Practice method	Mindfulness-based self-breathing
Type of practice	Bring one’s attention to breathing and concentrate on the natural flow of inhalation and exhalation.
Implementation steps	Sit comfortably with your eyes lightly closed. Pay attention to the natural sensations of your breath in the body. Follow the breath through full cycles, from the beginning of an inhalation to the end of an exhalation, and continue to the next cycle.. If your mind wanders due to internal and external stimuli and you lose awareness of the breath, gently return your attention to breathing.
Intervention period	Four weeks
Practice time (per session)	10 min
Time of practice	Any time of the day that is convenient for the participant (consistent timing throughout the intervention period is recommended).
Location of practice	Any location convenient for the participant (consistent location throughout the intervention period is recommended).
Approach	Daily practice is the ideal. However, intermittent continuation is acceptable if daily practice is difficult.

**Table 2:** Characteristics of participants.

Variable	Sub-Categories	N	%
Sex	Female	20	90.9
	Male	2	9.1
Age	20–29	16	72.7
	30–39	2	9.1
	40–49	2	9.1
	50–59	2	9.1
Employment status	Yes	9	40.9
	No (University students)	13	59.1
Subjective stress level	High	2	9.1
	Somewhat high	6	27.3
	Neither	6	27.3
	Somewhat low	6	27.3
	Low	2	9.1
Intervention implementation rate (out of 28 days)	Interruption for more than 3 days	5	22.7
	Interruption for less than 3 days	17	77.3

**Table 3:** The implementation rate of the mindfulness-based self-breathing intervention by each participant during the 28 days.

Participant No.	1st Wk.	2nd Wk.	3rd Wk.	4th Wk.	Mean
1	100	100	85.7	100	96.4
2	100	100	100	100	100
3	100	100	100	100	100
4	85.7	100	85.7	100	92.9
5	85.7	71.4	85.7	71.4	78.6 ^1^
6	100	100	100	100	100
7	85.7	100	71.4	71.4	82.1 ^1^
8	100	100	100	100	100
9	100	100	100	100	100
10	100	85.7	100	100	96.4
11	100	100	100	100	100
12	100	100	100	100	100
13	85.7	71.4	85.7	85.7	82.1 ^1^
14	85.7	100	100	85.7	92.89
15	100	100	100	100	100
16	85.7	100	100	100	96.4
17	85.7	85.7	85.7	85.7	85.7 ^1^
18	100	100	100	100	100
19	100	100	100	100	100
20	100	100	85.7	100	96.4
21	85.7	71.4	57.1	85.7	75.0 ^1^
22	100	100	100	100	100
Mean	94.8	94.8	92.9	94.8	94.3

^1^ Interruption of more than 3 days.

**Table 4:** Total and sub-factor FFMQ scores before (pre), during, and after (post) the mindfulness-based self-breathing intervention.

FFMQ Score	Median (IQR)			*p*-Value (Effect Size r)		
	Pre	During	Post	Pre–During	During–Post	Pre–Post
Total score	115.5 (111.25–125.75)	123 (109.75–138.75)	129 (117.75–147.75)	0.683(0.08)	0.002 * (0.22)	<0.001 * (0.30)
Sub-factor scores						
Observing	24 (19.75–28)	25 (21.5–29.5)	28 (20.25–32.5)	0.211 (0.12)	0.775 (0.07)	0.010 * (0.19)
Nonjudging	24.5 (19.75–32.5)	27 (21.75–30.25)	29 (24.0–32)	1.00 (0.04)	0.016 * (0.18)	0.086 (0.14)
Nonreactivity	19 (18–21.25)	21 (19–23.25)	25 (20.5–26.25)	0.395 (0.10)	0.025 * (0.17)	<0.001 * (0.27)
Acting with awareness	25.5 (21–28.25)	27 (24.25–32)	28.5 (24.5–30.25)	0.395 (0.10)	1.00 (0.05)	0.058 (0.16)
Describing	23.5 (19.75–28.25)	24 (15–31)	27.5 (15–32.25)	1.00 (0.04)	0.071 (0.15)	0.010 * (0.19)

Data presented as median and interquartile ranges. FFMQ, the Japanese version of the Five Facet Mindfulness Questionnaire. The asterisk indicates a *p*-value lower than 0.05. Differences between the time points were calculated using the Friedman test, followed by the Dunn–Bonferroni correction. The effect size was calculated using Pearson’s product–moment correlation coefficient.

**Table 5:** Differences in total and sub-factor FFMQ scores between the study participants with continuous (n = 17) or intermittent (n = 5) practice at each measurement time.

FFMQ Score	Group	Median (IQR)			*p*-Value (Effect Size r)		
		Pre	During	Post	Pre	During	Post
Total score	Continuous	113 (109–131)	118 (110–135)	128 (117–143)	0.880 (0.03)	0.446 (0.18)	0.319 (0.23)
	Intermittent	118 (112–121)	127 (119–144)	130 (128–152)			
Sub-factor scores							
Observing	Continuous	24 (19–26)	25 (22–26)	25 (18–31)	0.058 (0.41)	0.085 (0.38)	0.101 (0.36)
	Intermittent	28 (27–30)	32 (26–33)	32 (31–36)			
Nonjudging	Continuous	25 (20–34)	27 (21–29)	29 (24–32)	0.704 (0.08)	0.543 (0.14)	0.704 (0.09)
	Intermittent	24 (20–27)	27 (25–32)	28 (27–32)			
Nonreactivity	Continuous	19 (18–22)	21 (20–22)	25 (23–26)	0.101 (0.36)	0.319 (0.23)	0.704 (0.08)
	Intermittent	18 (15–19)	19 (17–23)	24 (18–26)			
Acting with awareness	Continuous	25 (21–29)	28 (22–32)	29 (25–30)	0.762 (0.08)	0.820 (0.06)	0.940 (0.03)
	Intermittent	26 (25–26)	26 (26–31)	28 (26–30)			
Describing	Continuous	24 (21–28)	24 (16–31)	27 (22–32)	1.000 (0.08)	0.543 (0.13)	0.401 (0.18)
	Intermittent	23 (20–27)	25 (23–28)	28 (26–30)			

Data presented as median and interquartile ranges. FFMQ, the Japanese version of the Five Facet Mindfulness Questionnaire. The continuous group includes participants with continued practice or interruptions for less than 3 days. Differences between the two groups were calculated using the Mann–Whitney U test. The effect size was calculated using Pearson’s product–moment correlation coefficient.

## Data Availability

The data used and/or analyzed in this study are available from the corresponding author upon reasonable request and within ethical constraints.
